# 

**Published:** 1902-06

**Authors:** 


**NOTICE.—This JOURNAL and THE INTERNATIONAL JOURNAL OF SURGERY one year for $1.00 cash, to new subscribers. No out-of-town Checks accepted unless 10c. is added for cost of cashing.**

THE First Annual Meeting of the Georgia State Sociological Society will be held in Atlanta, Tuesday, Wednesday and Thursday, June 24, 25 and 26, 1902.

An interesting program of papers, discussions and committee reports will soon be completed.

It is the object of this Society to improve the social conditions of our State, and develop a public sentiment in favor of a higher moral and intellectual life.

Every person interested in the future welfare and advancement of the State should be interested in this movement. All such are especially urged to attend the meeting, join the Society, and aid in the work.

The following permanent committees will report during the meeting :

1. Crime and its Prevention, Dr. S. G. C. Pinckney, Chairman.
2. Insanity, Including Mental Degeneracy, Dr. T. O. Powell, Chairman.

3. Alcoholism and its Prevention, Dr. W. B. Parks, Chairman.

4. Tuberculosis, its Development, Prevention and Control, Dr. J. N. Brawner, Chairman.

5. Diseases and Sanitary Conditions, Dr. J. L. Hiers, Chairman.

6. Education. Public Schools and Child Labor, —————— Chairman.

7. The Negro Problem, its Influence on the White Race, Rev. C. B. Wilmer, Chairman.

Papers on Sociological subjects of interest have been promised by the following:

Out of the State:—Dr. Daniel R. Brower, Chicago, Ill.; Dr. Geo. H. Price, Nashville, Tenn.; Dr. J. C. LeGrand, Birmingham, Ala.; Dr. R. C. Bankston, Birmingham, Ala.

In the State:—Attorney F. S. Key Smith, Rome; Dr. R. H. Taylor, Griffin; Dr. J. L. Hiers, Savannah; Dr. E. J. Spratlin, Forsyth; Rev. A. R. Holderby, Atlanta; Rev. J. C. Solomon, Atlanta; Rev. C. B. Wilmer, Atlanta; Dr. S. G. C. Pinckney, Atlanta; Rev. C. A. Langston, Atlanta; Attorney Shepard Bryan, Atlanta; Rev. Alonzo Monk, Atlanta; Dr. J. C. Jarnagin, Atlanta; Dr. A. W. Stirling, Atlanta; Dr. W. B. Parks, Atlanta; Dr. J. N. Brawner, Atlanta; Dr. R. R. Kime, Atlanta.

Other papers may be added before the meeting, as others are to be heard from, giving their titles.

Railroad rates of one and one-third fare on certificate plan have been secured.

A complete program will soon be published.

All other organizations within the State organized for the protection or benefit of mankind, are invited to send delegates to the meeting in June.

R. R. KIME, President,
Atlanta, Ga.

**This Journal and Atlanta Semi-weekly Journal one year for $1.00 cash in advance. No checks. Strictly to new subscribers.**

**This Journal and Atlanta Semi-weekly Journal one year for $1.00 cash in advance. No checks. Strictly to new subscribers.**

